# Ex vivo localization and immunohistochemical detection of sentinel lymph node micrometastasis in patients with colorectal cancer can upgrade tumor staging

**DOI:** 10.1186/1746-1596-7-71

**Published:** 2012-06-22

**Authors:** Fu-Long Wang, Fang Shen, De-Sen Wan, Zhen-Hai Lu, Li-Ren Li, Gong Chen, Xiao-Jun Wu, Pei-Rong Ding, Ling-Heng Kong, Zhi-Zhong Pan

**Affiliations:** 1 State Key Laboratory of Oncology in South China; Department of Colorectal Surgery, Cancer Center, Sun Yat-sen University, 651 Dongfengdong Road, Guangzhou, Guangdong, 510060, P. R. China

**Keywords:** Colorectal carcinoma, Sentinel lymph node, Micrometastasis, Prognosis

## Abstract

**Background:**

It is not clear if sentinel lymph node (SLN) mapping can improve outcomes in patients with colorectal cancers. The purpose of this study was to determine the prognostic values of ex vivo sentinel lymph node (SLN) mapping and immunohistochemical (IHC) detection of SLN micrometastasis in colorectal cancers.

**Methods:**

Colorectal cancer specimens were obtained during radical resections and the SLN was identified by injecting a 1% isosulfan blue solution submucosally and circumferentially around the tumor within 30 min after surgery. The first node to stain blue was defined as the SLN. SLNs negative by hematoxylin and eosin (HE) staining were further examined for micrometastasis using cytokeratin IHC.

**Results:**

A total of 54 patients between 25 and 82 years of age were enrolled, including 32 males and 22 females. More than 70% of patients were T3 or above, about 86% of patients were stage II or III, and approximately 90% of patients had lesions grade II or above. Sentinel lymph nodes were detected in all 54 patients. There were 32 patients in whom no lymph node micrometastasis were detected by HE staining and 22 patients with positive lymph nodes micrometastasis detected by HE staining in non-SLNs. In contrast only 7 SLNs stained positive with HE. Using HE examination as the standard, the sensitivity, non-detection rate, and accuracy rate of SLN micrometastasis detection were 31.8% (7/22), 68.2% (15/22), and 72.2%, respectively. Micrometastasis were identified by ICH in 4 of the 32 patients with HE-negative stained lymph nodes, resulting in an upstaging rate 12.5% (4/32). The 4 patients who were upstaged consisted of 2 stage I patients and 2 stage II patients who were upstaged to stage III. Those without lymph node metastasis by HE staining who were upstaged by IHC detection of micrometastasis had a significantly poorer disease-free survival (p = 0.001) and overall survival (p = 0.004).

**Conclusion:**

Ex vivo localization and immunohistochemical detection of sentinel lymph node micrometastasis in patients with colorectal cancer can upgrade tumor staging, and may become a factor affecting prognosis and guiding treatment.

**Virtual slides:**

The virtual slide(s) for this article can be found here: http://www.diagnosticpathology.diagnomx.eu/vs/1350200526694475.

## Introduction

Colorectal cancer is the most common malignancy in men and women, and the fourth leading cause of cancer-related death worldwide [1]. The status of lymph nodes in colorectal carcinoma is the most important prognostic factor for recurrence and overall survival (OS), and also largely determines whether adjuvant chemotherapy should be given [2]. Approximately 70-90% of patients with no lymph node involvement will survive 5 years, whereas only 40% of those with lymph node metastases will survive for that length of time. However, about 30% of patients with node-negative colon cancer staged by standard pathologic techniques experience recurrent locoregional or distant metastases within 5 years [1,2]. One of the possible reasons for the variations in outcome among patients with node-negative disease is that there is inaccurate or incomplete nodal staging [3].

All lymph nodes within resected specimens should be examined after surgery to obtain an accurate N stage; however, small lymph nodes with metastases are likely to be missed during conventional gross pathological examination. Study has shown that > 70% of the positive lymph nodes are < 2 mm [4]. The benchmark of 12 as a minimum acceptable number of lymph nodes obtained during colorectal cancer resection has been adopted as a quality measure for surgical practice by many organizations. Even so, two large population-based studies found that only 37-49% of patients with colon cancer receive an adequate lymph node evaluation [5-7]. Techniques such as fat clearance are used to increase the yield of lymph nodes, but these methods are time-consuming and not used routinely.

Several studies have reported nodal micrometastasis in colorectal cancer by examination methods such as serial sectioning, immunohistochemistry (IHC), or reverse transcriptase-polymerase chain reaction (RT-PCR). However, it is not clear if these findings indicate a worse prognosis [5,8-11]. In addition, these ultrastaging techniques are too costly and time-consuming for use on the 12 or more lymph nodes in every colorectal cancer specimen.

The sentinel node (SLN) is the first lymph node to receive lymphatic drainage from a tumor. In concept, the SLN has the highest chance of harboring tumor cells because they directly drain from the tumor. The concept of SLN allows focused examination on just a few nodes at greatest risk for metastatic involvement. If lymph node staging can be upgraded by focusing on detection of a few SLNs using conventional and immunohistochemical techniques, better clinical outcomes may be expected. With successful application of SLN detection technique in breast cancer [12-14], investigators have attempted to apply this technique in colorectal cancer. Many studies have demonstrated the feasibility of applying SLN detection techniques in colorectal cancer; however, the detection rate, sensitivity, and false negative rate vary considerably [1,3,4,15-26]. Moreover, as yet only a few studies have shown that survival of colorectal cancer patients can be influenced by the application of the SLN technique [18,19,27].

The purpose of this study was to determine the prognostic value of ex vivo sentinel lymph node (SLN) mapping and immunohistochemical detection of SLN micrometastasis in colorectal cancers.

## Materials and methods

### Patients

A total of 54 patients with surgery-naive colorectal cancers who received radical operations at the Department of Abdominal Surgery, Sun Yat-sen University Cancer Center between March and October of 2003 were included in this study. All patients had proven colorectal adenocarcinoma by preoperative pathological biopsy examination, and all received clinical examination, chest X-ray, abdominal ultrasound, colonoscopy, and thoracoabdominal computed tomography (CT) scans. Patients with multiple primary colorectal tumors, tumor local excision, severe local infiltration, surgery in tumor lymphatic drainage areas, emergency operations, non-en bloc resections, and M1 patients preoperatively or intraoperatively were excluded from this study. Informed consent was obtained from all patients, and this study was approved by the Institutional Review Board of our hospital.

### SLN mapping

Enrolled patients received standard radical operations per conventional procedures after routine preoperative preparations. SLN localization was performed on ex vivo specimens within 30 minutes or resection using 1% isosulfan blue solution (Sigma-Aldrich USA). The specimen was opened at the anti-mesenteric border and 0.5-2 ml (depending on the volume of the tumor) 1% isosulfan blue solution was injected submucosally and circumferentially around the tumor. The injection sites were then gently massaged for 5 minutes to push the tracer into the lymphatic vessels. The first node to stain blue was defined as the SLN.

### Pathologic examination

The SLNs were excised and processed separately. Thereafter, a minimum of 12 non-SLNs as recommended by the UICC/AJCC guidelines were dissected from the specimen. All lymph nodes were collected by the same surgeon. Nodal samples were fixed in formalin and embedded in paraffin. All lymph nodes were sectioned at least one section at 5 um thickness, and were separated for hematoxylin and eosin (HE) staining. SLNs that were determined negative by conventional pathological methods were further examined using cytokeratin (CK; monoclonal mouse anti-cytokeratin AE1/AE3; Abcam,1:100) IHC to identify micrometastasis. Sentinel nodes were examined using at least four multilevel sections at 250-um intervals. Positive lymph node micrometastasis was defined by identification of one or more CK-labeled positive cells in lymph nodes in which metastases were not detected by HE staining in combination with histological and cell morphological findings. All samples, including tumors, nodes, HE-stained, and IHC-stained sections were evaluated separately by two experienced pathologists. If different results were obtained, a third pathologist reviewed the specimens and slides to make the final determination.

SLNs and non-SLNs which were positive for micrometastasis by HE staining were categorized as N+, negative lymph nodes were categorized as N0, and SLNs with micrometastasis detected by IHC staining only were categorized as N0(i+). All treatment decisions were based upon the results of standard HE histopathological staging. Patients with stage II and III disease were given fluorouracil-based adjuvant chemotherapies.

### Patient follow-up

All patients were followed-up through clinic visits. Follow-up included clinical examination, carcinoembryonic antigen (CEA) level, chest X-ray, and abdominal ultrasound every 3–6 months for 2 years, then every 6–12 months. Patients with multiple polyps in the colon received colonoscopies annually, and those with vessel invasion or poor differentiation also received CT scans annually.

### Statistical analysis

Overall survival (OS) duration was calculated from the date when a patient was enrolled in the study to the date of death or last follow-up visit. Disease-free survival (DFS) duration was defined as the interval between the date of enrollment in the study and the date recurrence or metastasis was identified.

Data were presented as mean ± standard deviation for continuous variables and number (percentage) for categorical variables. Kaplan-Meier survival curves were calculated, and the difference in survival statuses among the three groups (N+, N0, and N0(i+)) was tested by the Breslow estimation method. A Cox proportional hazard model was used to determine the factors associated with survival status, recurrence, and metastasis. Factors with p < 0.1 revealed by univariate Cox proportional hazard model analysis, age, and sex were then included in further multiple analyses in order to establish the final model. The forward stepwise method was applied when establishing the multiple Cox proportional model. Statistical significance was defined as p < 0.05. All statistical analyses were performed using PASW software (version 18.0, IBM SPSS Inc., Chicago, IL).

In addition, detection rate, accuracy, sensitivity, and false negative rate were reported for the examination of SLN localization. These indexes were determined in accordance of following formulas [20]:

Detection rate (%): (number of patients with successfully retrieved SLNs/number of patients enrolled) × 100.

Sensitivity (%) of lymph node micrometastasis: (number of patients with positive SLNs/number of patients with micrometastasis in any lymph node) × 100.

False-negative rate (%): 100 - sensitivity

Accuracy (%): (number of patients with correct nodal status predicted/number of patients enrolled) × 100.

Lymph node ratio: (number of positive lymph nodes/total number of lymph nodes) × 100.

## Results

### Patients and clinical characteristics

A total of 54 patients between 25 and 82 years of age were enrolled, including 32 males and 22 females. Patient demographic and clinical data are shown in Table 1. More than 80% of patients were stage T3 or above, about 86% of patients were stage II or III, and approximately 90% of patients had lesions grade II or above.

**Table 1:** Patient demographic and clinical data

**Variables**	**Statistics**
Age (y)	57.00 ± 13.82
Lymph node ratio	0.05 ± 0.10
Survival duration (months)	61.69 ± 17.20
DFS duration (month)	56.33 ± 23.38
Gender	
Male	32 (59.3)
Female	22 (40.7)
Location of tumor	
Colon	28 (51.9)
Rectum	26 (48.1)
Depth of invasion	
T1	2 (3.7)
T2	8 (14.8)
T3	21 (38.9)
T4	23 (42.6)
Stage	
I	7 (13.0)
II	25 (46.3)
III	22 (40.7)
Death	13 (24.1)
Recurrence or metastasis	14 (25.9)

DFS, disease free survival; HE, hematoxylin-eosin; SLN, sentinel lymph node.

Continuous variables were shown as mean ± standard deviation and categorical variables were presented as number (%).

### HE staining for micrometastasis detection

Sentinel lymph nodes were detected in all 54 patients. There were 32 patients in whom no lymph node micrometastasis were detected by HE staining (N0) and 22 patients with positive lymph nodes micrometastasis detected by HE staining (N+) in non-SLNs. In contrast only 7 SLNs stained positive with HE (Table 2). Using HE examination as the standard, the sensitivity, non-detection rate, and accuracy rate of SLN micrometastasis detection were 31.8% (7/22), 68.2% (15/22), and 72.2% (32 + 7 = 39/54), respectively.

**Table 2:** HE examination for micrometastasis in SLNs (N = 54)

**HE staining**	**Non-SLN**	**SLN**
Positive (N+)	22	7
Negative (N0)	32	47

HE, hematoxylin-eosin; SLN, sentinel lymph node.

Further examination for lymph node micrometastasis by ICH in patients who were HE(−) showed that 12 of the 47 patients were positive for micrometastasis in SLNs (Table 3). In those patients, 4 of 12 had non-SLNs that were HE-negative (N0(i+)), and 8 patients had non-SLNs that were HE-positive. The 4 patients who were upstaged consisted of 2 stage I patients and 2 stage II patients who were upstaged to stage III; thus the upstaging rate was 12.5% (4/32 of non-SLN HE(−) patients). Additionally, based on the assumption that the results of micrometastasis detection could be a reference in terms of stage, the accuracy rate was increased from 72.2% to 87% [(7 + 32 + 8)/54].

**Table 3:** **IHC staining for micrometastasis in patients with HE-negative SLNs** (n = 47)

**ICH staining**	**SLN**
Negative	35
Positive	12*

IHC, immunohistochemistry; HE, hematoxylin-eosin; SLN, sentinel lymph node.

*Patients with positive ICH staining, of whom 4 of 12 had HE-negative stained lymph nodes and 8 had HE-negative stained SLNs but HE-positive stained non-SLNs at primary HE staining.

### Survival analysis

Among the 54 patients, 4 patients with micrometastasis detected in SLNs by IHC staining only were upstaged (N0(i+), group A), 28 patients had negative HE and IHC staining (N0, group B), and the other 22 patients had positive HE staining (N+, group C). In groups A and B, two patients in each group had metastatic disease and died, and in group C, 10 had recurrent or metastatic disease and 9 died.

**Figure 1:**
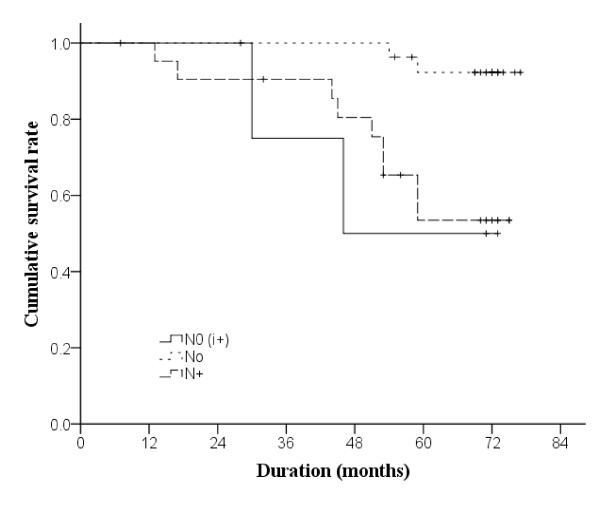
**Kaplan-Meier curves of overall survival in 54 patients (*n*=3 in group N0(i+),*n*=28 in group N0, Nand *n*=22 in group N+).** Breslow estimation method was used to test differences in survival among groups.

The Kaplan-Meier OS curve is shown in Figure 1. The survival status of the three groups were significantly different (p = 0.003). Regardless of upstaging status, all patients were alive 1 year after they participated in the study. At the third and the fifth year, the OS of group A was reduced to 75% and 50%, respectively; that of group B also declined to 96.3% and 92.3%, respectively; and that of group C were 90.5% and 53.5% respectively.

**Figure 2:**
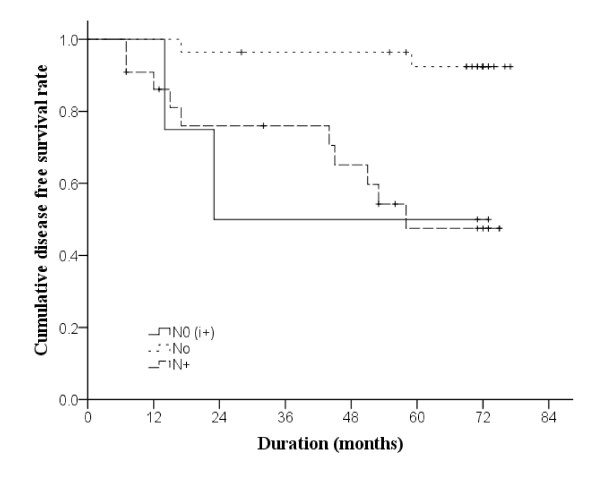
**Kaplan-Meier curves of disease-free survival in 54 patients (*n*=3 in group N0(i+),*n*=28 in group N0, and *n*=22 in group N+).** Breslow estimation method was used to test differences in disease-free survival among groups.

The Kaplan-Meier DFS curve is shown in Figure 2. In group A, the 1- and 3-year DFS was 100% and 50%, respectively, and no further change was noted. From 100% at the first year, the DFS rate of group B decreased to 96.4% and 92.4% at 3- and 5-years, respectively. In group C, the 1-year DFS rate was 86.1%, and decreased to 76.0% and 47.5% at 3- and 5-years, respectively. The three groups had statistically different DFS status (p = 0.001).

### Cox proportional hazard model

The results of Cox proportional hazard model with regard to survival are summarized in Table 4. The hazard of death was increased as the lymph node ratio increased (hazard ratio [HR] = 1.08, 95% confidence interval [CI]: 1.03-1.13, p = 0.003). Moreover, patients with an upstage of N0(i+) (HR = 10.28, 95% CI: 1.44-73.34, p = 0.020) and N + (HR = 7.93, 95% CI: 1.71-36.83, p = 0.008) had a higher hazard of death as compared with those with an upstage of N0. In addition, patients with positive HE staining were more likely to be dead (HR = 4.82, 95% CI: 1.48-15.70, p = 0.009). However, HE staining and lymph node ratio were dropped in the multivariate model; only the effect of upstaging was significant.

**Table 4:** Cox proportional hazard model for survival status

	**Univariate**	**Multivariate**
Age (y)	0.99 (0.95-1.03)	0.99 (0.96-1.04)
Lymph node ratio^†^	1.08 (1.03-1.13)*	-
Gender		
Female	Reference	Reference
Male	2.49 (0.69-9.06)	1.16 (0.28-4.81)
Location of tumor		
Colon	Reference	-
Rectum	1.06 (0.36-3.16)	-
Depth of invasion		
T1/T2	Reference	Reference
T3	2.15 (0.46-10.12)	3.9 (0.62-24.42)
T4	0.70 (0.12-4.17)	0.76 (0.11-5.36)
Stage		
I	Reference	-
II	0.81 (0.08-7.75)	-
III	3.67 (0.47-29.00)	-
Tumor grade		
I	Reference	-
II	0.93 (0.12-7.31)	-
III	0.81 (0.07-9.03)	-
Upstage		
N0(i+)	10.28 (1.44-73.34)*	20.32 (2.09-197.98)*
N0	Reference	Reference
N+	7.93 (1.71-36.83)*	10.06 (2.04-49.58)*
HE examination		
Negative	Reference	-
Positive	4.82 (1.48-15.70)*	-
SLN examination		
Negative	Reference	-
Positive	1.50 (0.33-6.84)	-

HE, hematoxylin-eosin; SLN, sentinel lymph node.

Dash denotes variables were not included in the final model.

* Indicates p < 0.05.

^†^ Ratio values were multiplied by 100.

Similar results were found for recurrence or/and metastasis (Table 5). In univariate analysis, significant factors were lymph node ratio, upstaging, and positive HE staining; whereas, once again upstage was the only significant factor left in the final model. The hazard of death in N0(i+) and N + patients was 8.07 (95% CI: 1.09-59.50, p = 0.041) and 7.87 (95% CI: 1.68-36.79, p = 0.009) times higher than those with N0 disease.

**Table 5:** Cox proportional hazard model for recurrence or/and metastasis

	**Univariate**	**Multiple**
Age (y)	0.98 (0.95-1.02)	1.00 (0.97-1.04)
Lymph node ratio^†^	1.06 (1.02-1.11)*	-
Gender		
Female	Reference	Reference
Male	1.75 (0.55-5.58)	2.19 (0.59-8.20)
Location of tumor		
Colon	Reference	-
Rectum	1.16 (0.40-3.30)	-
Depth of invasion		
T1 or T2	Reference	-
T3	2.23 (0.47-10.51)	-
T4	1.03 (0.19-5.63)	-
Stage		
I	Reference	-
II	0.83 (0.09-8.02)	-
III	4.60 (0.59-36.07)	-
Tumor grade		
I	Reference	-
II	0.95 (0.12-7.41)	-
III	0.69 (0.06-7.67)	-
Upstage		
N0(i+)	9.97 (1.40-71.07)*	8.07 (1.09-59.50)*
N0	Reference	Reference
N+	9.67 (2.10-44.47)*	7.87 (1.68-36.79)*
HE examination		
Negative	Reference	-
Positive	5.93 (1.84-19.12)*	-
SLN examination		
Negative	Reference	-
Positive	1.11 (0.25-4.96)	-

HE, hematoxylin-eosin; SLN, sentinel lymph node.

Dash denotes variables were not included in the final model.

* Indicates p < 0.05.

^†^ Ratio values were multiplied by 100.

## Discussion

Lymph node metastasis is one of the key factors of poor outcomes in patients with colorectal cancer. However, recurrence and metastasis rates are high in stage II colorectal cancer patients even without lymph node metastasis, and it has been reported that stage IIB patients have a poorer prognosis than stage III patients [11,21]. Inaccurate lymph node staging may be one of the reasons for the low survival rate. Methods examined to improve lymph node staging include increasing number of lymph nodes for postoperative examination and using micrometastasis detection; however, it is difficult to apply these techniques in clinical practice due to procedural complexity and costs.

Theoretically, SLN detection can help reduce understaging because the SLN is the first to receive drainage from the tumor. In our study, ex vivo SLN detection was used in 54 patients and we found an identification rate of 100% and 4 patients were upstaged. Our results are consistent with other studies that have shown SLN mapping results in a higher detection rate and upstaging when combined with immunohistochemical or RT-PCR techniques [1,16,17,20,22]. A prospective randomized study of SLN ultrastaging showed that SLNs were successfully identified in 82 of 84 patients (97.6%), and significant nodal upstaging occurred (38.7% to 57.3%, p = 0.019) [22]. Another prospective multicenter trial demonstrated that at least one SLN was identified in 268 of 315 enrolled patients (detection rate, 85%) and 21% (30 of 141) of the patients, classified as pN0 by routine histopathological examination, were found to have micrometastasis or isolated tumor cells in the SLN [20]. Interestingly, a study by Cheng et al. [28] of colorectal cancer patients and liver metastases showed that nuclear beta-catenin overexpression in metastatic lymph nodes was strongly associated with liver metastasis. Perin et al. [29] reported a case a 46-year-old female who underwent sigmoid colon resection for colon cancer who subsequently developed pathologically proven breast metastasis of the colon malignancy. Examination of the axillary SLN revealed metastasis consistent with the colon primary.

Despite the encouraging results, there remains controversy regarding false negative rates. Our false negative rate was 68.2%, higher than that reported by other authors, and our results indicated that the SLN did not predict overall nodal status. This is similar to the findings of a recent systematic review and meta-analysis performed by van der Pas et al. [1]. The authors included 52 studies with 3767 SLN procedures (78.6% colon carcinoma, 21.4% rectal carcinoma) and found a mean detection rate of 94% and a pooled sensitivity of 76%. Retter et al. [30] reported the results of 31 patients who received surgery for colon carcinoma in which *in vivo* SLN mapping was performed, and found that although the SLN was identified in 28 of the 31 patients, the false-negative rate to identify stage III disease was 66% and the accuracy was 14%. Finan et al. [31] also reported that ex vivo SLN mapping did not improve staging after proctectomy for rectal cancer. In contrast, van Schaik et al. [4] reported no false negative SLNs in 44 patients. The reasons for the high false-negative rates in some studies are unclear, but may possibly include the T stage, tumor location, and learning curve for SLN mapping. Thus, we believe that the cumulative data indicate that routine SLN detection alone cannot replace the conventional method that examines all dissected lymph nodes because a high false-negative rate tends to downgrade tumor staging such that some patients who may benefit from adjuvant chemotherapy will not receive it.

Although almost all studies analyzing SLN mapping for colorectal cancer demonstrate the detection of micrometastasis, the clinical significance of lymph node micrometastasis, particularly that identified solely by IHC staining, is unknown. There have been extensive studies on lymph node micrometastasis using IHC or RT-PCR techniques, and some reports have indicated that micrometastasis detection by ICH was associated with poor prognosis of colon cancers [8-10]. However, other studies have reported conflicting findings [5,11]. Dahl et al. [18] reported that only patients with metastatic lymph nodes detected directly or within a SLN died of metastatic disease. Saha et al. [19] demonstrated with a 2-year minimum follow-up of 153 patients who underwent SLN mapping, 7% had recurrences as compared with 25% of 162 patients with standard resection and nodal staging. Bilchik et al. [27] reported that no colon cancer patient with a negative SLN by HE and PCR had a recurrence at a mean follow-up of 25 months. Our results indicated that patients without lymph node metastasis by HE staining who are upstaged by ICH detection of micrometastasis have a significantly poorer OS and DFS. However, survival analysis showed no difference in OS and DFS between the stage III and stage N0(i+) groups. This may imply that patients with SLN micrometastasis have the same prognosis as patients with stage III disease, which suggests that focusing on the detection of SLN micrometastasis could become a useful factor in determining prognosis, although further study is warranted due to limited number of cases. In addition, in multivariate analysis upstaging by SLN micrometastasis identification was not an independent prognostic factor; the small number of study patients may be the reason.

The primary limitation of the study is the relatively small number of patients. However, the results do support those of other studies of the utility of SLN mapping and IHC detection of micrometastasis.

## Conclusions

Our findings indicate that SLN detection is feasible for colorectal cancers, and ex vivo localization and micrometastasis detection of SLNs in colorectal cancers can upgrade tumor staging. Though SNL examination in combination with IHC for the detection of micrometastasis can reduce the false-negative rate, the method is not suggested to replace the conventional N-staging method that examines all dissected lymph nodes. Moreover, ex vivo localization and micrometastasis detection of SLNs in colorectal cancers may become a factor affecting prognosis and guiding treatment.

## Competing interests

The author(s) declare that they have no competing interests.

## Authors’ contributions

F-LW, FS have made substantial contributions to conception and design, acquisition of data, analysis of data; involved in drafting the manuscript. D-SW, Z-HL, L-RL, GC, X-JW, P-RD, L-HK participated in the design of the study; involved in revising manuscript critically for important intellectual content. Z-ZP have made substantial contributions to conception and design; involved in revising it critically for important intellectual content. All authors read and approved the final manuscript.
